# Association between peripheral lymphocyte count and the mortality risk of COVID-19 inpatients

**DOI:** 10.1186/s12890-021-01422-9

**Published:** 2021-02-11

**Authors:** Saibin Wang, Yijun Sheng, Junwei Tu, Lanlan Zhang

**Affiliations:** 1 Department of Respiratory Medicine, Jinhua Municipal Central Hospital, Jinhua Hospital of Zhejiang University, No. 365, East Renmin Road, Jinhua, 321000 Zhejiang Province China; 2 Department of Nursing, Wuhan Fourth Hospital, PuAi Hospital of Tongji Medical College, Huazhong University of Science and Technology, No. 66, South Xuefu Road, Wuhan, 430032 Hubei Province China

**Keywords:** Coronavirus disease 2019, Lymphocyte count, Non-linear relationship, Mortality, Prediction

## Abstract

**Background:**

To explore the relationship between peripheral lymphocyte counts (PLCs) and the mortality risk of coronavirus disease 2019 (COVID-19), as well as the potential of PLC for predicting COVID-19 hospitalized patients death.

**Methods:**

Baseline characteristics, laboratory tests, imaging examinations, and outcomes of 134 consecutive COVID-19 hospitalized patients were collected from a tertiary hospital in Wuhan city from January 25 to February 24, 2020. Multiple regression analysis was used to analyze the relationship between the PLC at admission and mortality risk in COVID-19 patients and to establish a model for predicting death in COVID-19 hospitalized patients based on PLC.

**Results:**

After adjusting for potential confounding factors, we found a non-linear relationship and threshold saturation effect between PLC and mortality risk in COVID-19 patients (infection point of PLC: 0.95 × 10^9^/L). Multiple regression analysis showed that when PLCs of COVID-19 patients were lower than 0.95 × 10^9^/L, the patients had a significantly higher mortality risk as compared to COVID-19 patient with PLCs > 0.95 × 10^9^/L (OR 7.27; 95% CI 1.10–48.25). The predictive power of PLC for death in COVID-19 patients (presented as area under the curve) was 0.78. The decision curve analysis showed that PLC had clinical utility for the prediction of death in COVID-19 inpatients.

**Conclusions:**

PLC had a non-linear relationship with mortality risk in COVID-19 inpatients. Reduced PLCs (< 0.95 × 10^9^/L) were associated with an increased mortality risk in COVID-19 inpatients. PLCs also had a potential predictive value for the death of COVID-19 inpatients.

**Supplementary Information:**

The online version contains supplementary material available at 10.1186/s12890-021-01422-9.

## Background

Coronavirus disease 2019 (COVID-19) is a severe acute respiratory infectious disease caused by severe acute respiratory syndrome coronavirus 2 (SARS-CoV-2), and it has been designated by the World Health Organization as a pandemic [1–3]. The pandemic has appeared in many countries and regions around the world [3, 4]. As of January 19, 2021, more than 95,000,000 confirmed COVID-19 cases were reported worldwide, with more than 2,000,000 deaths [4]. COVID-19 has a high transmission rate, and some COVID-19 patients have rapidly progressed to severe and critical conditions and even death [5, 6].

Lymphocytes produced by lymphoid organs are the smallest white blood cells (WBCs) and are important cellular components of the immune response in the body. They are also the main executor of immune functions of the lymphatic system and the front-line defense against external pathogens in the body [7, 8]. In addition to the pathogenicity of the virus itself, the inflammatory response of the host is a key factor in SARS-CoV-2-induced lung injury [9]. The host innate and adaptive immune responses play an important role in resisting the progression and prognosis of COVID-19 [9], indicating the close relationship between peripheral lymphocyte counts (PLCs) and the occurrence and progression of COVID-19. Further insight into the possible relationship between PLCs and COVID-19 may help to improve the understanding of COVID-19 and enhance the ability to treat the disease. The aim of this study was to perform a retrospective cohort study to analyze the specific relationship between PLCs and the mortality risk of COVID-19 inpatients and to determine the potential of PLCs in predicting death in COVID-19 inpatients.

## Methods

### Study population

This study included consecutive COVID-19 patients treated at Wuhan Fourth Hospital (Wuhan, China) from January 25 to February 24, 2020. The hospital is a large-scale tertiary hospital, which was a designated hospital in Wuhan, China for the treatment of severe COVID-19 patients. The inclusion criteria for this study were as follows: (1) diagnostic criteria for COVID-19 patients that followed the *Diagnosis and Treatment Plan of Coronavirus Disease 2019* (trial edition 7) issued by the National Health Commission of the People’s Republic of China [6]; and (2) COVID-19 patients who were admitted to and received complete treatment processes at a ward of Wuhan Fourth Hospital. The exclusion criteria for this study were COVID-19 patients: (1) who did not receive treatment at Wuhan Fourth Hospital or those who were transferred to other Hospitals due to their good condition; and (2) who had a history of cancer, immunodeficiency disease, underlying hematological malignancies, or who had been receiving radiation exposure for more than two weeks. Patients on chronic corticosteroids therapy (except for patients using inhaled corticosteroid preparations, such as asthma and chronic obstructive pulmonary disease) or other immune suppressive therapies (e.g., post-transplant patients) were also excluded. The endpoint of our analysis was the mortality assessment of COVID-19 patients during hospitalization. In this study, patients who met any of the following were classified as severe COVID-19 patients: a. shortness of breath, with a respiratory rate ≥ 30 times/min; b. the oxygen saturation is ≤ 93% in the resting state; c. oxygenation index (partial pressure of oxygen in the arterial blood / fraction of inspired oxygen concentration) ≤ 300 mmHg (1 mmHg = 0.133 kPa); d. the lesions have progressed significantly in lungs (> 50% within 24–48 h in imaging); e. respiratory failure occurs and mechanical ventilation is required; and f. shock or combined with other organ failure need to transfer to the intensive care unit. COVID-19 patients who did not have the aforementioned presentations but had clinical manifestations such as fever and cough, with or without imaging manifestations of pneumonia were classified as moderate COVID-19.

This study adhered to the ethical principles of the Declaration of Helsinki and was approved by the Ethics Committee of Wuhan Fourth Hospital (No. KY2020-033–01). All study data were collected from an electronic medical record system, and written informed consent was waived by the Ethics Committee of Wuhan Fourth Hospital for emerging infectious diseases.

### Variable collection

#### Demographic information

Demographic information was collected, including age, gender, weight, smoking history, and underlying co-morbidities (e.g., heart disease, hypertension, diabetes mellitus, chronic kidney diseases, and stroke).

#### Clinical information

Clinical symptoms (e.g., respiratory symptoms, such as cough, sputum, and dyspnea; gastrointestinal symptoms, such as nausea and vomiting; and systemic symptoms, such as myalgia and fatigue) and vital signs were collected in this study. The disease classification (e.g., moderate and severe cases) [6] and the treatment outcomes (survival or non-survival) were recorded as well.

#### Laboratory test results

The peripheral WBC count, neutrophil count, PLC, hemoglobin, platelet count, albumin, blood glucose, alanine aminotransferase, aspartate aminotransferase, total bilirubin, creatinine, urea nitrogen, uric acid, creatine kinase (CK), creatine kinase isoenzymes (CK-MB), lactate dehydrogenases (LDH), C-reactive proteins, prothrombin time (PT), activated partial thromboplastin time, and D-dimer levels at admission were included in the data analysis.

### Statistical analyses

The baseline characteristics were described. The quantitative data were presented as mean ± standard deviation for variables with normal distribution or median [interquartile range] for variables with abnormal distribution, and the categorical data were presented as number and percentage. Comparisons between two groups were performed using independent sample *t* test or Kruskal–Wallis rank sum test, Pearson’s chi-square test, or Fisher’s exact test. Multicollinearity between covariates was tested. After adjusting for potential confounding factors, general additive model and multiple regression analysis were performed to assess the relationship between PLCs and mortality risk from COVID-19. Threshold effect analysis was used to find the infection point of the relationship between PLCs and mortality risk from COVID-19. The Kaplan–Meier curve was used to show the death of COVID-19 patients in two lymphocyte groups (i.e., above or below the infection point of PLC) within a month, and the log-rank test was used to assess whether there was a statistical significant difference in mortality between the two groups. Strategies for adjusting confounding variables were as follows [10]: Strategy I—determining the variables (age and gender) that needed to be adjusted based on clinical significance; Strategy II—including the variable in Strategy I, and variables that adding the covariate to the basic model or removing the covariate from the complete model affected the regression coefficient of “X (lymphocytes)” > 10% as well as variables whose regression coefficient for “Y (death of COVID-1)” had *P* < 0.1. By calculating the area under the curve (AUC) to evaluate the discrimination ability of PLCs in predicting the death of COVID-19, a nomogram was conducted to display the predictive model. Calibration was performed using the unreliability test. In addition, a decision curve analysis (DCA) was used to evaluate the clinical applicability of this predictive model. All analyses were performed using R (The R Foundation; https://www.r-project.org) software and Empower (X&Y solutions, Inc., Boston, MA; http://www.empowerstats.com). *P* < 0.05 was considered statistically significant in this study.

## Results

Among the 134 COVID-19 patients, the severe cases accounted for 46.27% (62/134). Male patients accounted for 44.78% (60/134), and there were 21 (15.7%, 95% confidence interval: 9.5%-21.8%) cases of death. The PLC of the non-survival group (mean value: 0.69 × 10^9^/L) was significantly lower than that of the survival group (mean value: 1.16 × 10^9^/L) as assessed by univariate analysis (*P* < 0.001). The mean age of the patients in the non-survival group (65 years) was significantly higher than that of the survival group (56 years) (*P* < 0.05). Statistical differences between the two groups also included clinical manifestations (dyspnea and arterial oxygen saturation on room air) at the time of admission and other laboratory tests included WBC, neutrophil count, platelets, uric acid, CK, CK-MB, LDH, C-creative protein, PT, and D-dimer (Table 1 and Additional file 1: Table S1).

**Table 1:** Baseline characteristics of the study participants

Variables	Outcomes of Patients		*P* value
	Survival (n = 113)	Non-survival (n = 21)	
Male, n (%)	47 (41.59)	13 (61.90)	0.086
Age (year)	56 ± 14	65 ± 10	0.004
Smoking, n (%)	9 (8.0)	1 (4.8)	0.608
Arterial oxygen saturation, (%)	97.1 ± 6.6	83.5 ± 14.4	< 0.001
COPD, n (%)	4 (3.6)	1 (4.8)	0.583
Hypertension, n (%)	34 (30.1)	3 (14.3)	0.625
Diabetes mellitus, n (%)	8 (7.1)	4 (19.0)	0.078
Heart disease, n (%)	12 (10.6)	3 (14.3)	0.625
Chronic kidney disease, n (%)	2 (1.8)	0 (0.0)	1.000
Stroke, n (%)	2 (1.8)	3 (14.3)	0.027
Fever (°C), n (%)			0.509
< 37.3	11 (9.7)	2 (9.5)	
37.3–38.0	31 (27.4)	6 (28.6)	
38.1–39.0	53 (46.9)	7 (33.3)	
> 39.0	18 (15.9)	6 (28.6)	
Cough n (%)	88 (77.9)	17 (81.0)	0.753
Sputum production, n (%)	32 (28.3)	5 (23.8)	0.671
Dyspnea, n (%)	64 (56.6)	16 (80.0)	0.049
Haemoptysis, n (%)	2 (1.8)	2 (10.5)	0.100
***Laboratory tests***			
WBC (× 10^9^/L)	5.07 ± 1.99	8.14 ± 4.77	0.003
Lymphocyte count (× 10^9^/L)	1.16 ± 0.53	0.69 ± 0.52	< 0.001
Platelets (× 10^9^/L)	212 ± 96	169 ± 103	0.041
C-reactive protein (mg/L)	17.9 (6.2–43.2)	78.0 (42.9–119.4)	< 0.001
PT (S)	11.7 ± 4.2	14.0 ± 5.1	0.026
D-dimer (mg/L)	0.5 (0.3–1.1)	1.6 (0.3–8.5)	0.027
Albumin (g/L)	36.8 ± 6.7	35.7 ± 9.5	0.491
Uric acid (mmol/L)	259 ± 89	311 ± 117	0.024
LDH (IU/L)	220 (178–285)	337 (241–581)	0.002
CK (IU/L)	101.0 (60.1–196.0)	199.0 (92.0–453.3)	0.006
CK-MB (IU/L)	9.0 (5.2–12.7)	17.4 (12.0–34.7)	< 0.001
***Lesions on chest CT scan, n (%)***			0.574
Unilateral lung	12 (10.81)	1 (6.25)	
Bilateral lungs	99 (89.19)	15 (93.75)	

Data are presented as n (%), median (IQR), or mean ± SD. *COPD* chronic obstructive pulmonary disease, *WBC* white blood cell, *PT* prothrombin time, *LDH* lactate dehydrogenases, *CK* creatine kinase, *CK-MB* creatine kinase isoenzymes, *CT* computed tomography

**Fig. 1:**
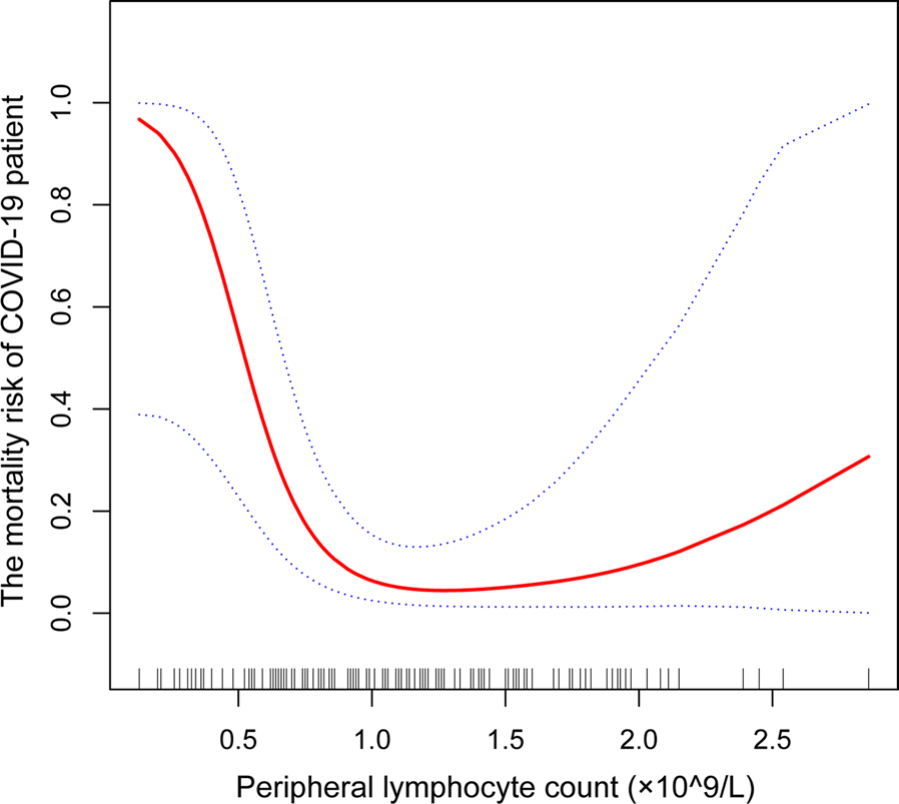
Smooth curve fitting shows a non-linear relationship between PLCs and the mortality risk of COVID-19 after adjusting the potential confounding factors (gender, age, smoking, diabetes mellitus, heart rate at admission, serum albumin, CK, CK-MB, uric acid, PT, and D-dimer). Dotted lines represent the upper and lower 95% confidence interval. *PLC* peripheral lymphocyte count, *COVID-19* coronavirus disease 2019, *CK* creatine kinase, *CK-MB* creatine kinase isoenzymes, *PT* prothrombin time

**Fig. 2:**
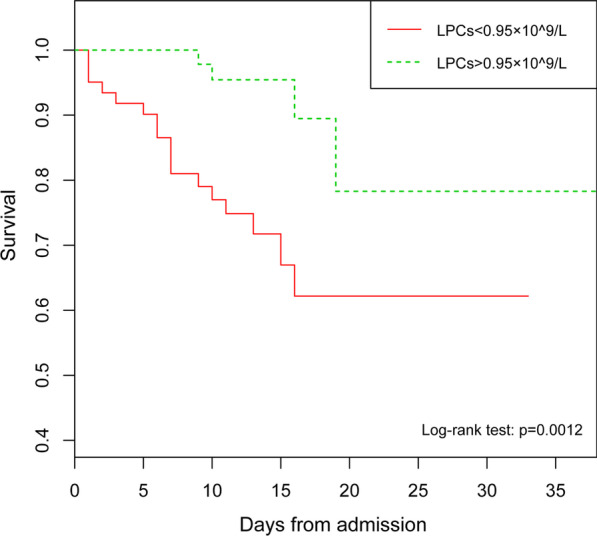
Kaplan–Meier curve. Estimated 1-month survival rate of COVID-19 patients with PLCs higher or lower 0.95 × 10^9^/L. *COVID-19* coronavirus disease 2019, *PLC* peripheral lymphocyte count

According to Strategy II for adjusting confounding variables, in addition to gender and age, we also adjusted variables selected in the covariate screening, including history of smoking, diabetes mellitus, heart rate at admission, serum albumin, CK, CK-MB, uric acid, PT, and D-dimer. A non-linear relationship was observed between the PLCs and mortality risk from COVID-19 in smooth curve fitting (Fig. 1). Further analysis showed that there was a threshold effect of PLCs on the relationship between PLCs and mortality risk of COVID-19 (Table 2). After adjusting for the potential confounding variables, the threshold of PLC was observed at 0.95 × 10^9^/L. We further divided the COVID-19 patients into two groups, with the 0.95 × 10^9^/L lymphocyte threshold as the cut-off value. In the multiple regression analysis, our results showed that regardless of adjusting for confounding variables or adjusting confounding variables according to different strategies (Strategy I or II), the mortality risk of COVID-19 was significantly higher in the patients with PLCs < 0.95 × 10^9^/L than the patients with PLCs > 0.95 × 10^9^/L (*P* < 0.05) (Table 3). Specifically, the patients who had lower levels of PLCs (< 0.95 × 10^9^/L) suffered from a 6.27-fold (Model II) higher risk of death than those with PLCs > 0.95 × 10^9^/L. Kaplan–Meier analysis showed in COVID-19 patients with PLCs levels exceeding the threshold value (0.95 × 10^9^/L) were associated with higher rates of survival than PLCs levels below the threshold value at one month (Fig. 2).

**Table 2:** Threshold effect of PLC on the mortality risk of COVID-19 in piecewise linear regression

Inflection point of PLC	The mortality risk of COVID-19^a^	
	OR (95% CI)	*P* value
< 0.95 × 10^9^/L	0.00 (0.00, 0.05)	0.0041
> 0.95 × 10^9^/L	4.59 (0.35, 59.80)	0.2442
Log-likelihood ratio test		0.001

*PLC* peripheral lymphocyte count, *COVID-19* coronavirus disease 2019, *CK* creatine kinase, *CK-MB* creatine kinase isoenzymes, *PT* prothrombin time

^a^Adjust for: gender, age, smoking, diabetes mellitus, heart rate at admission, serum albumin, CK, CK-MB, uric acid, PT, and D-dimer

**Table 3:** Multiple regression analysis of PLC on the mortality risk of COVID-19

PLC	Crude OR (95% CI) p-value	Model I^a^ OR (95% CI) p-value	Model II^b^ OR (95% CI) p-value
> 0.95 × 10^9^/L	Ref.	Ref.	Ref.
< 0.95 × 10^9^/L	6.66 (2.10, 21.11) < 0.01	5.33 (1.63, 17.47) < 0.01	7.27 (1.10, 48.25) < 0.05

^a^Adjust strategy-I adjusted for: gender and age

^b^Adjust strategy-II adjusted for: gender, age, smoking, diabetes mellitus, heart rate at admission, serum albumin, CK, CK-MB, uric acid, PT, and D-dimer

*PLC* peripheral lymphocyte count, *COVID-19* coronavirus disease 2019, *OR* odds ratio, *CI* confidence interval, *CK* creatine kinase, *CK-MB* creatine kinase isoenzymes, *PT* prothrombin time

**Fig. 3:**
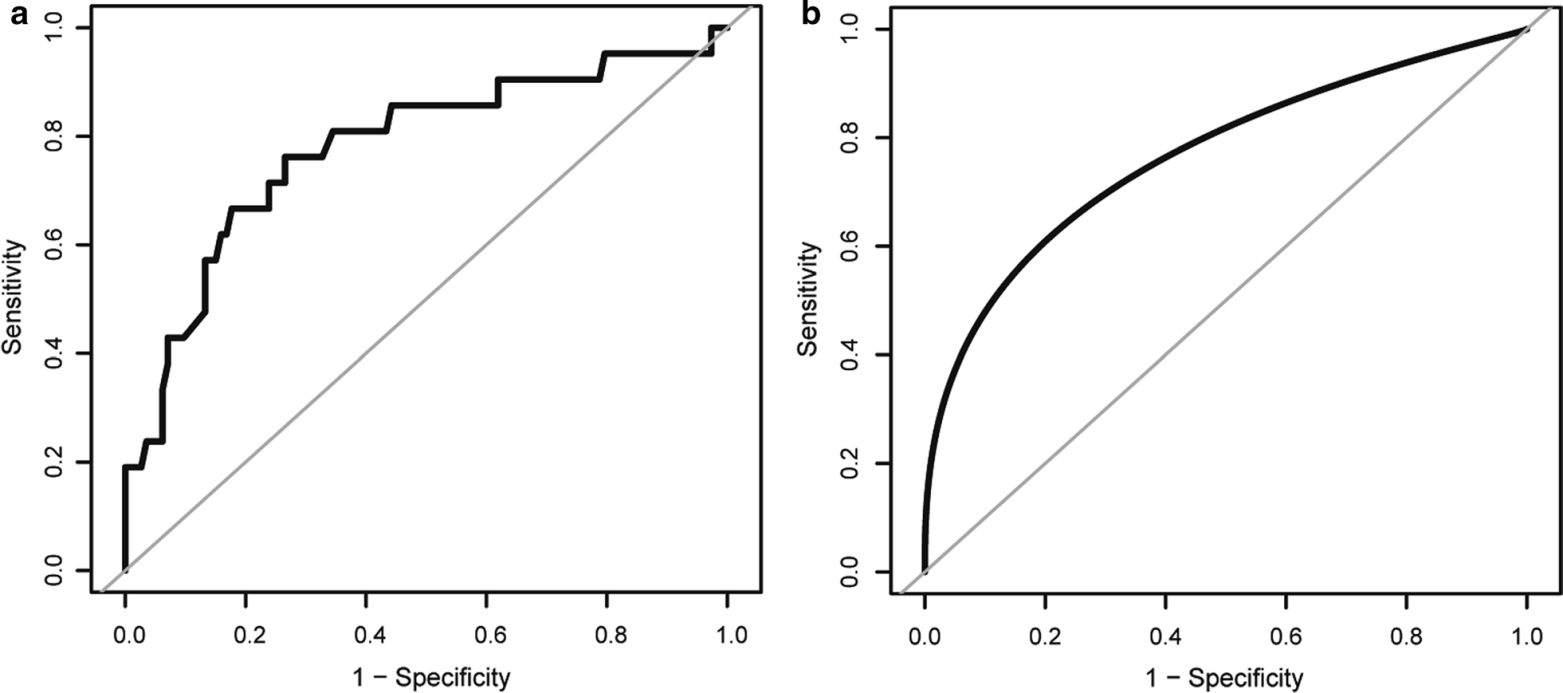
The discriminatory ability of PLC on admission in predicting the mortality risk of COVID-19 patients. The AUC for the predictive model is 0.78 (95% CI 0.66–0.90) (**a**), and for the internal validation using bootstrap method (resampling times: 500) was 0.77 (95% CI 0.64–0.87) (**b**). *PLC* peripheral lymphocyte count, *COVID-19* coronavirus disease 2019, *AUC* area under the curve, *CI* confidence interval

With regard to the discrimination ability of PLC on admission in predicting the mortality risk of COVID-19 patients, the AUC was 0.78 (Fig. 3a). The internal validation (bootstrap resampling times = 500) output AUC was 0.77 (Fig. 3b). With a cut-off value of 0.77 × 10^9^/L, the prediction of death from COVID-19 was as follows: 73.5% specificity, 76.2% sensitivity, 73.9% accuracy, and 94.3% negative predictive value. In addition, a *P*-value of 0.720 with an Emax value of 0.212 and Eavg value of 0.037 was yielded in the unreliability test, suggesting that the model was well calibrated.

**Fig. 4:**
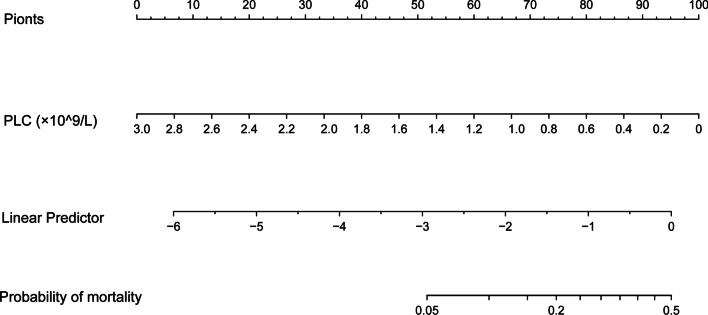
Nomogram of the risk of mortality. First row: point assignment of the PLC; second row: PLC on admission; the lowest row: prediction of the mortality risk of COVID-19 patients. *PLC* peripheral lymphocyte count, *COVID-19* coronavirus disease 2019

**Fig. 5:**
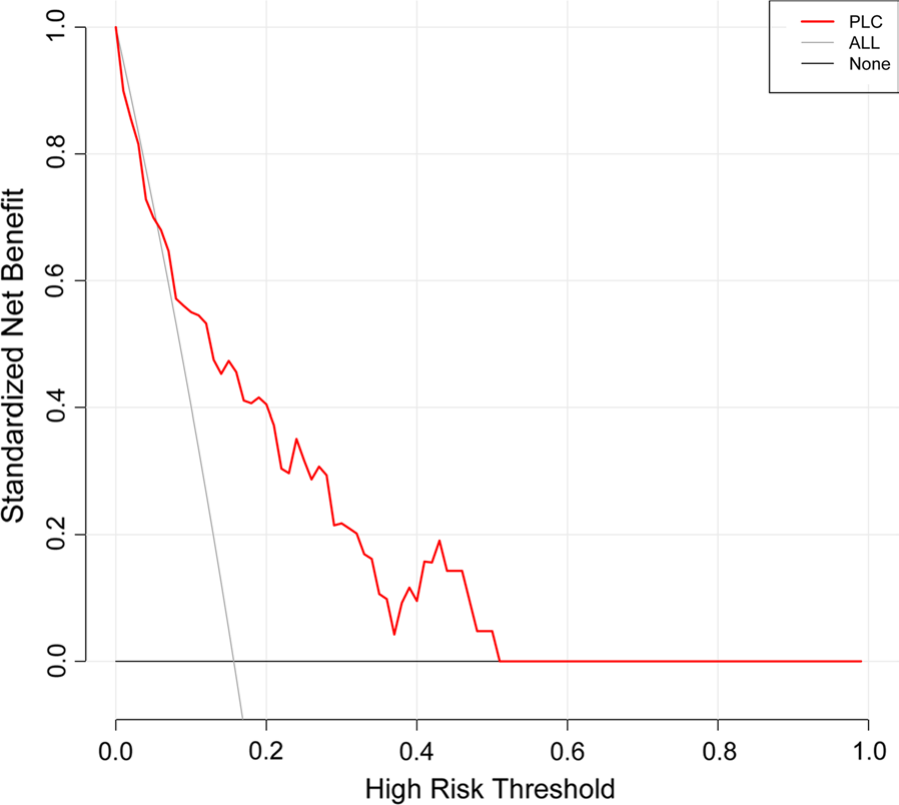
DCA of the predictive model. The red solid line represents the predictive model. The DCA indicates that when the threshold probability is less than 51%, application of PLC in predicting the death of COVID-19 patients would add clinical decision net benefit compared with either the treat-all or the treat-none strategies

To facilitate clinical application, a nomogram was constructed to show the prediction of death from COVID-19 based on the PLCs (Fig. 4). Moreover, the results of the DCA showed that PLC at admission had clinical utility in predicting death from COVID-19 (i.e., when the threshold of the mortality risk of COVID-19 was < 51%, PLC was able to predict the mortality risk in COVID-19 patients such that further clinical decisions yielded a net benefit) (Fig. 5).

## Discussion

This study explored the relationship between PLC and the mortality risk of COVID-19 and showed that there was a non-linear relationship and threshold saturation effect between the PLC and mortality risk of COVID-19. The infection point of the relationship occurred when the PLC was equal to 0.95 × 10^9^/L. When the PLC was less than 0.95 × 10^9^/L, the mortality risk from COVID-19 in patients significantly increased. In addition, we evaluated the value of the PLC for predicting the mortality risk of COVID-19 and showed that the PLC had potential predictive value, which should facilitate clinical decisions.

COVID-19 emerged at the end of 2019 and is a serious acute respiratory infection. It is highly contagious and has spread to many countries worldwide [1–5]. Fever, dry cough, myalgia, and fatigue are common clinical manifestations in COVID-19 patients, while flu-like symptoms and gastrointestinal symptoms are relatively rare [1, 11–13]. As of January 19, 2021, COVID-19 has caused more than 2,000,000 deaths [4]. Among COVID-19 patients, without distinguishing the disease severity, the reported mortality rate was between 0.9% and approximately 28% [14–16]. In this study, we found that the mortality rate of COVID-19 patients reached 15.7%, which was primarily because the patients in this study originated from a medical unit that mainly treated severe COVID-19 patients. This also showed that COVID-19 has a high mortality risk in severe cases. Importantly, during the early stage of COVID-19, the clinical manifestations of some severe and critically ill patients may not be obvious, but it quickly progresses to acute respiratory distress syndrome, septic shock, metabolic acidosis, coagulopathy, and even death [6]. Hence, exploring the risk factors of COVID-19 and establishing a predictive model of COVID-19 mortality are important for clinical treatment.

The intensity of the innate and adaptive immune response is closely related to the occurrence, development and prognosis of COVID-19 [9]. Lymphocytes are the main executors of adaptive immune functions [7, 8]. Previous studies have shown that the severity of COVID-19 may be related to PLC levels [12, 15–19]. However, the specific relationship between the two has not been clearly elucidated. This study retrospectively analyzed the clinical data in the patients with COVID-19 and showed a non-linear relationship and threshold saturation effect between PLC and the mortality risk from COVID-19. Patients with PLCs < 0.95 × 10^9^/L had approximately sevenfold mortality risk as compared to patients with PLCs > 0.95 × 10^9^/L. This relationship still existed after adjusting for potential confounding factors, suggesting that reduced PLC was an independent risk factor for death from COVID-19. In addition, the lymphocyte count has been used by several predictive models as one of the predictors of the diagnosis and prognosis of COVID-19 infection [20]. Here, we further studied the value of PLC alone in predicting death from COVID-19 by establishing a predictive model, which had a discrimination capacity of 0.78. In addition, to further explain whether our model had value in a clinical practice, we conducted a DCA and showed that the clinical application of our model was expected to achieve clinical benefits. For example, when the threshold of mortality risk from COVID-19 was set at 20% (i.e., when the patients were predicted to have a mortality risk > 20%, intervention measures were given to the patients), application of our predictive model in 100 COVID-19 patients would achieve clinical benefits in 40 COVID-19 patients, and the remaining 60 COVID-19 patients would have no harm from using this predictive model (i.e., compared with the strategy of taking intervention measures for all patients or taking non-intervention measures for all, the net benefit of making clinical decisions based on the prediction results of the predictive model was 40%). Therefore, PLCs in predicting death from COVID-19 had potentially good clinical application value.

However, this study did have some limitations. First, although we used multiple adjustment strategies to adjust for potential confounding factors, the study design of the retrospective analysis made it difficult to completely avoid confounding factors (e.g., the incubation period of the disease and time to hospital presentation) and selection bias. Second, this study was a single-center study, and there may be differences in the timing and treatment measures of COVID-19 patients in different countries and regions, which might lead to different PLCs at the time of admission. Therefore, the practicability of our predictive model needs to be externally validated in other centers for further clarity. Third, our model may not be suitable for a population of COVID-19 patients who are mainly diagnosed and treated as having moderate COVID-19 because nearly half of the cases participating in this study were severe COVID-19 patients. The reported mortality rate in our study does not represent the final mortality rate in the hospital because some patients still receiving treatment were not included in the analysis. Nevertheless, this study explained for the first time the specific relationship between PLC and death from COVID-19 and also explored the value of PLCs predicting death from COVID-19.

## Conclusions

This study showed that low PLC (< 0.95 × 10^9^/L) was closely related to an increased mortality risk of COVID-19 inpatients. In addition, PLC demonstrated value in predicting the mortality risk of COVID-19. The results from this study may help assess the conditions and prognosis of COVID-19 patients, thereby assisting the management and treatment of COVID-19 inpatients.

## Supplementary Information

**Additional file 1: Supplemental Table S1**. Other baseline characteristics of the study participants.

## Data Availability

The datasets used and/or analyzed during the current study are available from the corresponding author on reasonable request.

## Abbreviations

PLC: Peripheral lymphocyte count
COVID-19: Coronavirus disease 2019
SARS-CoV-2: Severe acute respiratory syndrome coronavirus 2
CK: Creatine kinase
CK-MB: Creatine kinase isoenzymes
LDH: Lactate dehydrogenases
PT: Prothrombin time
AUC: Area under the curve
DCA: Decision curve analysis
