# Supplementary material for: Association between peripheral lymphocyte count and the mortality risk of COVID-19 inpatients
Source: BMC Pulm Med. 2021 Feb 11;21:55. doi: 10.1186/s12890-021-01422-9 (PMC7877317; doi:10.1186/s12890-021-01422-9)
Supplement: Supplementary file 1 — Additional file 1: Supplemental Table S1. Other baseline characteristics of the study participants. [file 12890_2021_1422_MOESM1_ESM.docx]

**Table 1s**. **Other baseline characteristics of the study participants**

| **Variables** | **Outcomes of Patients** | | \| P-value \| \| --- \| \| |
| --- | --- | --- | --- | --- |
|  | **Survival (n = 113)** | **Non-survival (n = 21)** |  |
| Weight (kg) | 62.0 (52.0-72.8) | 78.0 (59.0-105.5) | 0.166 |
| SBP (mmHg) | 129.5 ± 17.9 | 131.8 ± 20.3 | 0.285 |
| DBP (mmHg) | 79.0 (71-88) | 78.0 (70-87) | 0.612 |
| **Signs and symptoms** |  |  |  |
| Gastrointestinal symptoms, n (%) | 40 (35.7) | 3 (16.7 | 0.111 |
| Myalgia, n (%) | 27 (25.2) | 3 (21.4) | 0.757 |
| Fatigue, n (%) | 75 (67.6) | 15 (78.9) | 0.321 |
| Headache, n (%) | 14 (13.1) | 3 (17.6) | 0.611 |
| **Laboratory Tests** |  |  |  |
| Neutrophil count (×10^9^/L) | 3.44 ± 1.78 | 6.19 ± 4.37 | 0.002 |
| Hemoglobin (g/L) | 126 ± 18 | 129 ± 23 | 0.520 |
| APTT (S) | 27.8 ± 6.9 | 30.7 ± 11.0 | 0.113 |
| ALT (IU/L) | 24.0 (15.0-43.0) | 43.0 (19.0-71.0) | 0.054 |
| AST (IU/L) | 29.0 (22.0-40.0) | 50.6 (32.0-71.0) | 0.139 |
| Total bilirubin (umol/L) | 11.8 ± 5.6 | 15.0 ± 8.3 | 0.116 |
| Creatinine (umol/L) | 62.6 (51.5-76.0) | 81.1 (66.1-99.2) | 0.669 |
| BUN (mmol/L) | 4.7 (3.6-5.7) | 5.0 (3.7-8.8) | 0.113 |
| Blood glucose (mmol/L) | 6.4 ± 2.1 | 7.4 ± 4.5 | 0.102 |

Data are presented as n (%), median (IQR), or mean ± SD. SBP, systolic blood pressure; DBP, diastolic blood pressure; APTT, activated partial thromboplastin time; ALT, alanine aminotransferase; AST, aspartate aminotransferase; BUN, Blood urea nitrogen; LDH, lactate dehydrogenases.
